# Professional Secrets

**Published:** 1874-01

**Authors:** 


Professional Secrets.—During the last week the question was raised by one of the profession, as to whether he was bound to reveal what he called professional secrets. We can understand perfectly well that a physician might feel a dislike to communicate anything given to him in confidence, and so would any other man. The being called to see a patient makes him the recipient of very many secrets, personal secrets, family secrets. There are scores of delicate matters within the circle, that no one outside the circle, but the physician, can know or even suspect. The honor of a family may be necessarily confided to him. The fortune and even the life of a patient may be dependent upon the physician's tongue, as well as upon his medicinal treatment. Outside of the sick room, he knows nothing of what transpires in it. The delirium which reveals secrets, he does not hear; or if he does, forgets. All this is the rule. But every rule has its exceptions. One great exception to this rule is the rule of the law. He who goes upon oath before the grand jury or before the court, swears that he will "tell the truth, the whole truth and nothing but the truth," and imprecates Divine vengeance upon his head, if he violates his word. Even here, an exception comes in, and he is allowed to withhold his testimony, provided he says, still under oath, that it will criminate himself. This is the only exception that we know of.

There seems to be an impression with many medical men that they have no right to reveal what they call professional secrets. As we before intimated, they have no right to be tattlers and scandal-mongers, as some are whom we know of, but there the matter of professional etiquette ceases. It is true, a medical man may feel a diffidence, a hesitation about revealing a matter which came to him in his daily business. He may caution a patient not to tell what he does not wish to have repeated, not to expose what he desires to have kept a secret; but what he must under oath reveal. If he chooses, he may balance his friendship for a patient against his oath, and calculate the value of that patient's confidence in comparison with perjury, or with the punishment to be inflicted for contempt of court.

